# Supplementary material for: Identification of transmissible proteotoxic oligomer-like fibrils that expand conformational diversity of amyloid assemblies
Source: Commun Biol. 2021 Aug 5;4:939. doi: 10.1038/s42003-021-02466-7 (PMC8342456; doi:10.1038/s42003-021-02466-7)
Supplement: Supplementary file 2 — Supplementary Information [file 42003_2021_2466_MOESM2_ESM.pdf]

Supplementary Information

Identification of transmissible proteotoxic oligomer-like fibrils that expand conformational diversity of amyloid assemblies

Phuong Trang Nguyen[1,2], Ximena Zottig[1,2], Mathew Sebastiao[1,2], Alexandre Arnold[1,2], Isabelle Marcotte[1,2] and Steve Bourgault*[1,2]

[1]Department of Chemistry, Université du Québec à Montréal, Montreal, Canada, H3C 3P8,

[2]Quebec Network for Research on Protein Function, Engineering, and Applications, PROTEO, Quebec, Canada

[*]Corresponding author: Prof. Steve Bourgault, Canada Research Chair in Chemistry of Biological Nanoassemblies, Department of Chemistry, Université du Québec à Montréal, C.P. 8888, Succursale Centre-Ville, Montréal (Québec), H3C 3P8, Canada, Telephone: (514) 987-3000 (5161); e-mail: bourgault.steve@uqam.ca

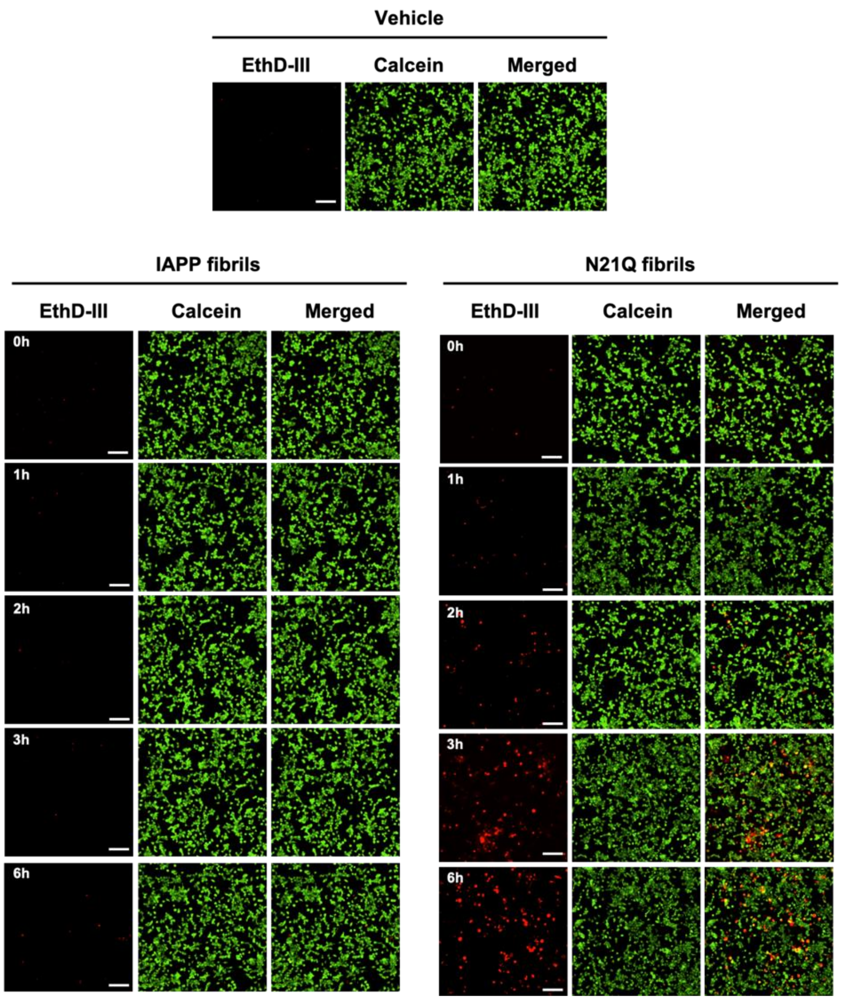

Supplementary Fig. 1 Cytotoxicity of IAPP and N21Q fibrils measured by the LIVE/DEAD assay. Representative fluorescence microscopy images showing the distribution of lived (green) and dead (red) INS-1E cells after treatment with 50 µM fibrils for 0 h to 6 h. Scale bar: 100 µm. Fibrils were assembled from freshly dissolved monomerized peptides incubated under quiescent conditions for 48 h at a concentration of 150 µM in 20 mM Tris-HCl buffer, pH 7.4.

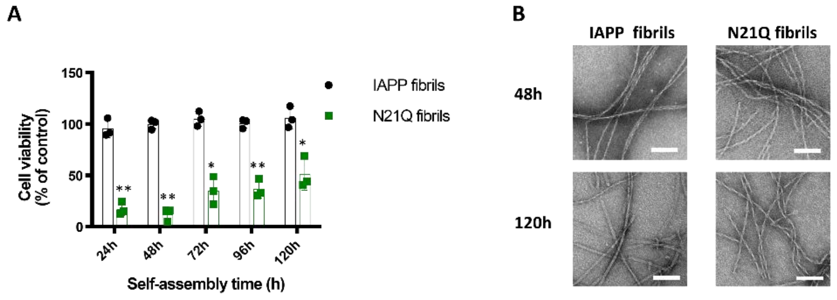

Supplementary Fig. 2 Fibril toxicity over extended self-assembly periods. (A) Cell viability of INS-1E treated with 50 µM of WT and N21Q fibrils assembled for different time, ranging between 24 h and 120 h. Data represent mean ± S.E.M of at least four individual experiments performed in triplicate. (B) TEM images of IAPP and N21Q fibrils after 48 h and 120 h of self-assembly time. Scale bar: 100 nm. (A,B) Fibrils were assembled from freshly dissolved monomerized peptides incubated under quiescent conditions at a concentration of 150 µM in 20 mM Tris-HCl buffer, pH 7.4.

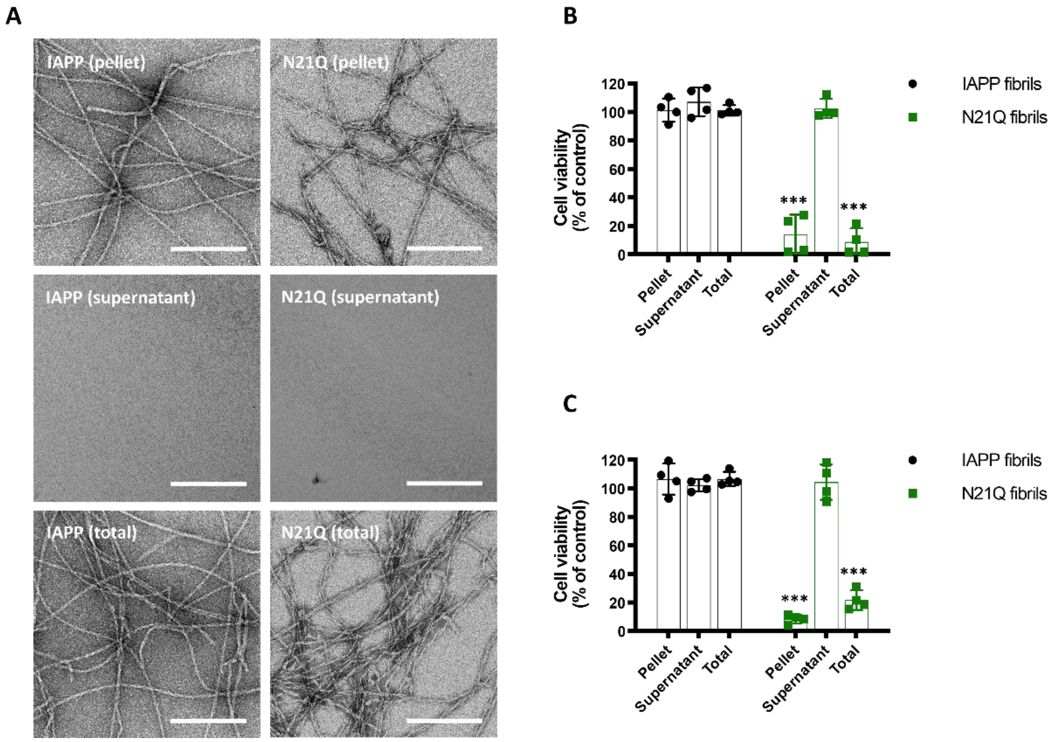

Supplementary Fig. 3 Cytotoxicity of isolated IAPP and N21Q fibrils. (A) Assemblies recovered after centrifugation were imaged by TEM. (B,C) Cytotoxicity of isolated fibrils (B) without sonication and with (C) 30 min sonication. Data represent mean ± S.E.M of at least four individual experiments performed in triplicate. INS-1E cells were incubated with 50 µM fibrils. (A,B,C) Fibrils were assembled from freshly dissolved monomerized peptides incubated under quiescent conditions for 48 h at a concentration of 150 µM in 20 mM Tris-HCl buffer, pH 7.4. Fibrils were isolated by two successive centrifugations at 35 000g for 45 min at 4 °C.

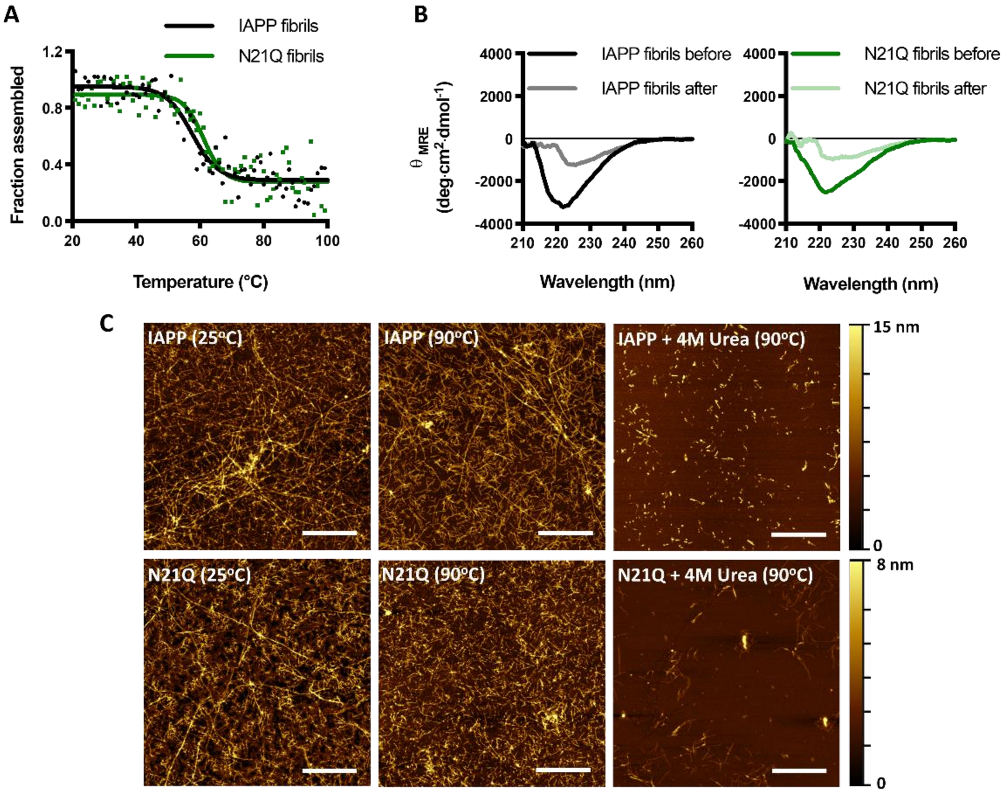

Supplementary Fig. 4 Thermal stability of IAPP and N21Q fibrils. (A) Representative thermal denaturation curve of IAPP and N21Q fibrils monitored by CD spectroscopy with ellipticity at 218 nm. IAPP fibrils showed a T_m of 58.1 ± 0.2 ^oC and N21Q fibrils showed a T_m of 61.3 ± 0.5^oC. (B) Representative CD spectra of IAPP and N21Q fibrils at 25 ^oC and 100 ^oC in presence of 4 M urea. (C) AFM images of IAPP and N21Q fibrils before (25°C) and after thermal unfolding (90°C) in absence, or in presence, of 4 M urea. (A-C) Fibrils were assembled from freshly dissolved monomerized peptides incubated under quiescent conditions for 48 h at a concentration of 150 µM in 20 mM Tris-HCl buffer, pH 7.4. Fibrils were diluted to 50 µM immediately before analysis.

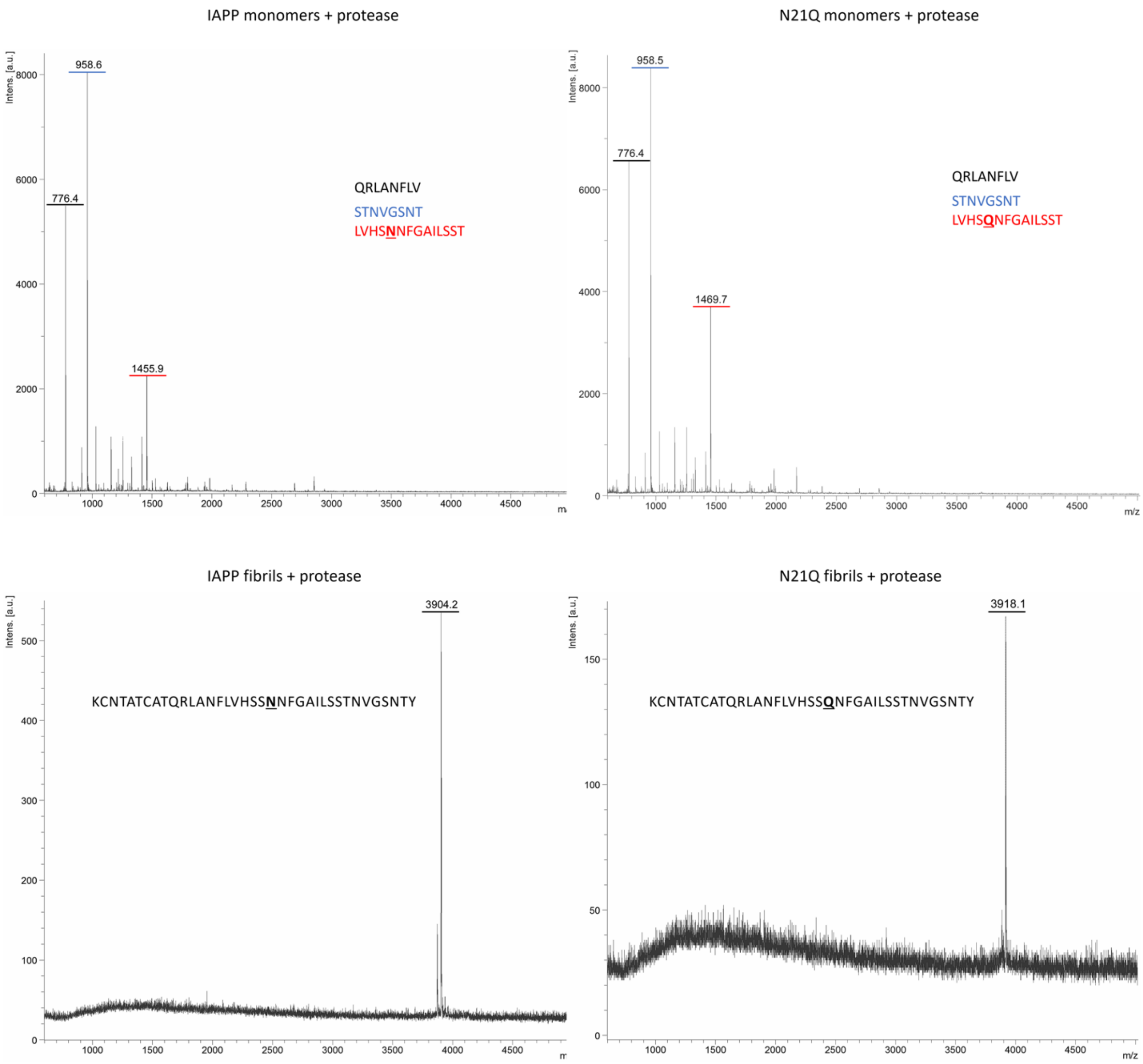

Supplementary Fig. 5 Stability of IAPP and N21Q fibrils against proteinase K proteolysis. MALDI-TOF mass spectrometry analysis of monomers and fibrils after treatment with proteinase K. Fibrils were assembled from freshly dissolved monomerized peptides incubated under quiescent conditions for 48 h at a concentration of 150 µM in 20 mM Tris-HCl buffer, pH 7.4. The assemblies were incubated with 120 U/ml of proteinase K for 1 h before MALDI-TOF analysis.

	Average		Median	
	**IAPP**	**N21Q**	**IAPP**	**N21Q**
**Fibril length**	1.42 ± 0.36 µm	1.08 ± 0.17 µm	2.55 µm	1.76 µm
**Fibril height**	5.98 ± 2.62 nm	3.12 ± 1.24 nm	6.11 nm	3.07 nm
**Young's modulus**	1.47 ± 0.78 GPa	1.21 ± 0.39 GPa	2.16 GPa	1.20 GPa

Supplementary Fig. 6 Mean and median values of length, height and Young's modulus of IAPP and N21Q fibrils. Peptide were assembled from freshly dissolved monomerized peptides and incubated for 48h at 150 µM in 20 mM Tris-HCl buffer, pH 7.4, under quiescent conditions before analysis of by AFM. At least 3000 fibrils per peptide were quantified.

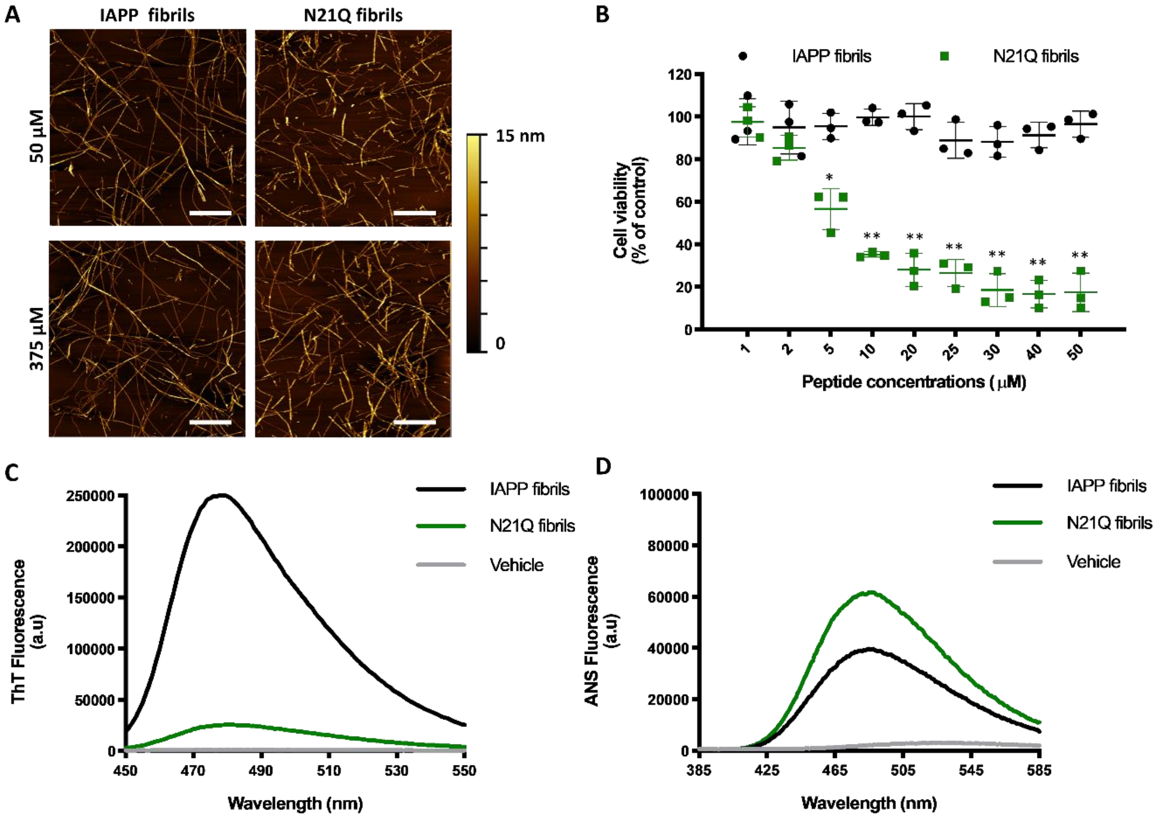

Supplementary Fig. 7 Characterization of IAPP and N21Q fibrils after lyophilization. (A) Representative AFM images of IAPP and N21Q fibrils resuspended in water after lyophilization. (B) INS-1E cells were incubated for 24 h with reconstituted fibrils, with concentrations ranging between 1 to 50 µM. Data represent mean ± S.E.M of at least four experiments performed in triplicate. (C) ThT fluorescence of reconstituted fibrils. The emission spectra of ThT (40 µM) was recorded from 450 nm to 550 nm with excitation at 440 nm. (D) Surface hydrophobicity of reconstituted fibrils measured by ANS fluorescence. The emission spectra of ANS (100 µM) was recorded from 385 nm to 585 nm with excitation at 355 nm. (A-D) Fibrils were assembled at 375 µM in 20 mM Tris-HCl, pH 7.4 for 48 h at room temperature under quiescent conditions. The resulting fibrils were recovered by ultracentrifugation at 100 000 g for 45 min at 4 °C. The pellet was re-suspended in nanopure water and lyophilized. Lyophilized aliquot were reconstituted in water to reach a concentration of 50 µM or 150 µM before analysis.

A

Residue	Carbon	δ IAPP (ppm)	δ N21Q (ppm)	δ RC (ppm)	Δδ IAPP (ppm)	Δδ N21Q (ppm)
**Ala13**	CO	171.8	173.0	175.1	-3.3	-2.1
	Cα	48.1	48.5	49.8	-1.7	-1.3
	Cβ	19.1	20.0	16.4	2.7	3.6
**Phe23**	CO	169.8	173.4	173.1	-3.3	0.3
	Cα	53.2	53.4	55.0	-1.8	-1.6
	Cβ	40.2	40.3	36.9	3.3	3.4
**Val32**	CO	170.9	173.0	173.6	-2.7	-0.6
	Cα	57.8	57.2	59.5	-1.7	-2.3
	Cβ	31.9	32.0	30.2	1.7	1.8
	Cγ	18.0	18.9	18.4	-	-

B

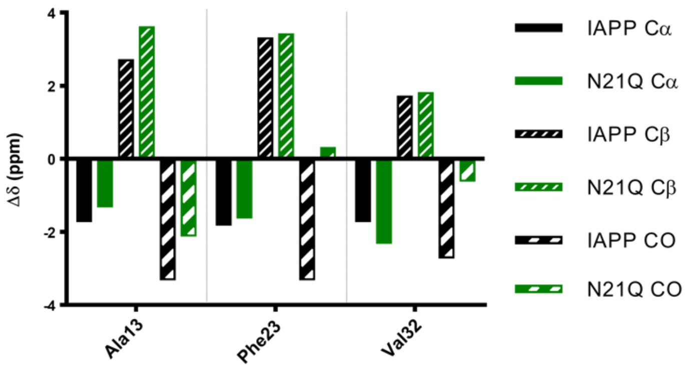

Supplementary Fig. 8 Secondary ^{13}C chemical shifts of Ala13, Phe23 and Val32. (A) Table of ^{13}C chemical shifts (Δδ) for Cα, Cβ, and CO of labelled residues calculated as $\Delta\delta = \delta_{obs} - \delta_{RC.}$ (B) Summarized of ^{13}C chemical shifts (Δδ) of Ala13, Phe23 and Val32 in IAPP and N21Q fibrils. (A,B) Fibrils were assembled at 750 µM in 20 mM Tris-HCl, pH 7.4 for 48 h at room temperature under quiescent conditions. The resulting fibrils were recovered by ultracentrifugation at 100 000 g for 45 min at 4 °C. Supernatant was removed and the fibril pellet was re-suspended in nanopure water and lyophilized.

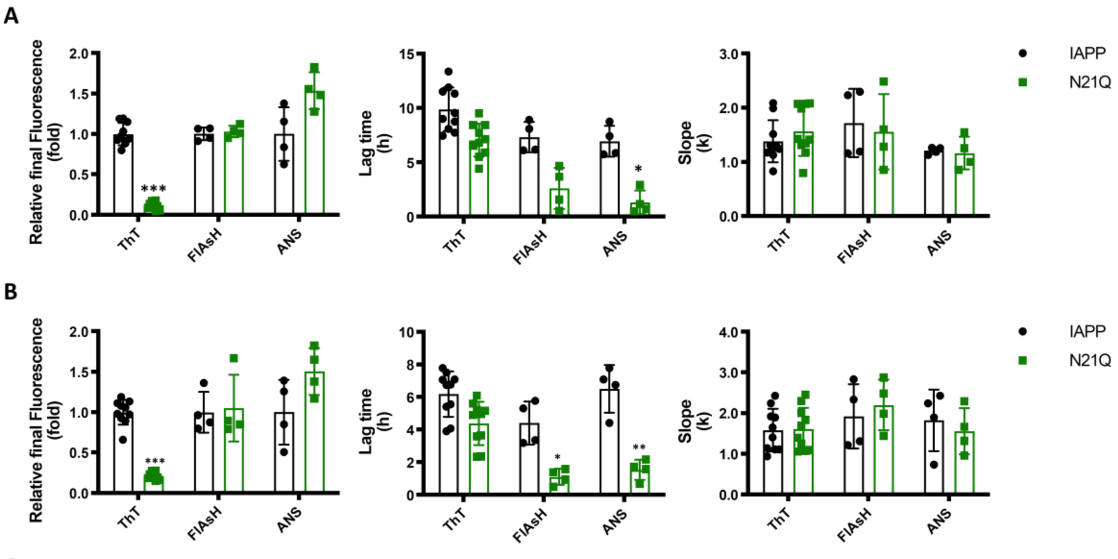

C

		Relative fluorescence (x fold)		Lag time (h)		Slope (k)	
		IAPP	N21Q	IAPP	N21Q	IAPP	N21Q
12.5 µM	ThT	1.000 ± 0.136	0.115 ± 0.047	9.871 ± 2.019	7.046 ± 1.533	1.384 ± 0.386	1.569 ± 0.456
	FlAsH	1.000 ± 0.080	1.031 ± 0.072	7.327 ± 1.394	2.612 ± 1.892	1.720 ± 0.630	1.557 ± 1.401
	ANS	1.000 ± 0.334	1.536 ± 0.229	6.952 ± 1.431	1.317 ± 1.089	1.210 ± 0.062	1.163 ± 0.304
25 µM	ThT	1.000 ± 0.153	0.211 ± 0.046	6.193 ± 1.385	4.388 ± 1.323	1.582 ± 0.524	1.613 ± 0.522
	FlAsH	1.000 ± 0.251	1.052 ± 0.414	4.410 ± 1.325	1.104 ± 0.487	1.924 ± 0.791	2.201 ± 0.622
	ANS	1.000 ± 0.402	1.506 ± 0.290	6.514 ± 1.463	1.537 ± 0.623	1.824 ± 0.760	1.557 ± 0.569

Supplementary Fig. 9 Kinetics of amyloid formation. Relative final fluorescence, lag time and slope determined by fitted sigmoidal curve at (A) 12.5 µM and (B) 25 µM peptide using ThT, FlAsH and ANS fluorescence. (C) Kinetic parameters monitored by ThT, FlAsH and ANS. (A,B,C) Peptides were incubated at 12.5 µM or 25 µM under quiescent conditions in 20 mM Tris-HCl buffer, pH 7.4 in the presence of ThT (40 µM) or FlAsH (0.5 µM) or ANS (50 µM). Fluorescence of ThT (Ex 440 nm, Em 485 nm), FlAsH (Ex 508 nm, Em 533 nm) or ANS (Ex 355 nm, Em 480 nm) was measured every 10 min. Data represent mean ± S.D. of at least four experiments performed in triplicate.

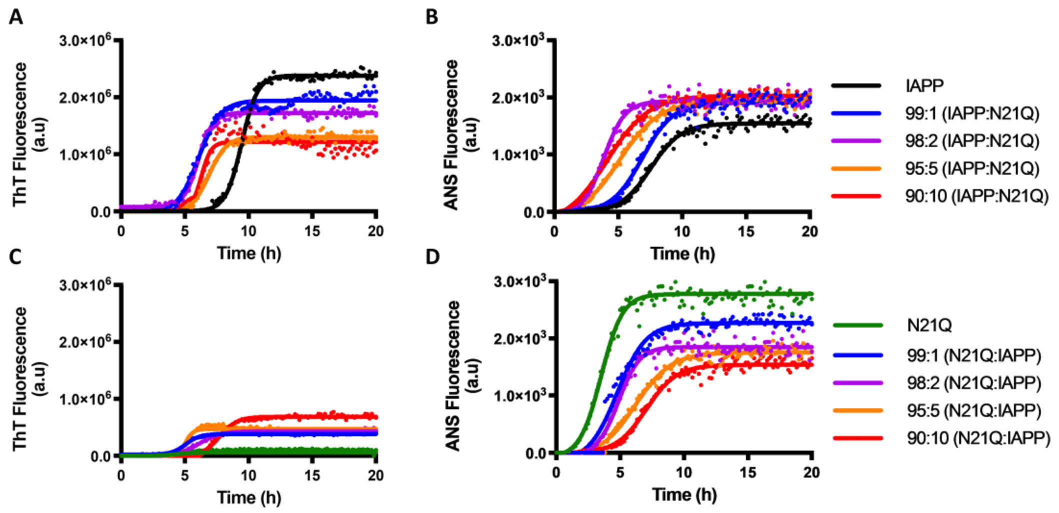

E

		IAPP	IAPP : N21Q				N21Q	N21Q : IAPP			
			99:1	98:2	95:5	90:10		99:1	98:2	95:5	90:10
Relative fluorescence (x fold)	ThT	1.00 ± 0.22	0.81 ± 0.06	0.72 ± 0.05	0.55 ± 0.03	0.51 ± 0.07	0.22 ± 0.02	0.16 ± 0.06	0.18 ± 0.05	0.19 ± 0.01	0.28 ± 0.05
	ANS	1.00 ± 0.15	1.24 ± 0.88	1.25 ± 0.21	1.24 ± 0.12	1.31 ± 0.50	1.92 ± 0.35	1.78 ± 0.31	1.45 ± 0.64	1.37 ± 0.42	1.20 ± 0.47
Lag time (h)	ThT	7.87 ± 1.12	5.12 ± 1.03	4.45 ± 0.92	3.91 ± 1.01	3.31 ± 1.10	4.05 ± 1.03	5.07 ± 1.10	5.08 ± 1.23	6.12 ± 1.15	7.75 ± 0.95
	ANS	7.15 ± 1.23	6.90 ± 0.12	4.12 ± 0.21	3.87 ± 0.09	1.92 ± 0.52	2.13 ± 1.02	3.76 ± 0.09	4.79 ± 0.06	5.19 ± 0.12	6.06 ± 0.09
Slope (k)	ThT	1.67 ± 0.29	1.17 ± 0.02	1.74 ± 0.70	1.44 ± 0.36	2.62 ± 0.09	1.15 ± 0.46	1.75 ± 0.13	1.17 ± 0.17	3.34 ± 0.42	1.45 ± 0.26
	ANS	1.01 ± 0.12	0.83 ± 0.09	1.20 ± 0.03	0.97 ± 0.07	0.62 ± 0.23	1.17 ± 0.39	0.88 ± 0.20	1.22 ± 0.07	0.73 ± 0.11	0.89 ± 0.03

Supplementary Fig. 10 Kinetics of co-assembly. (A-D) Kinetics of self-assembly of IAPP/N21Q mixtures monitored by ThT (A,C) and ANS fluorescence (B,D). Monomerized peptides were incubated at total concentration of 12.5 µM under quiescent conditions in 20 mM Tris-HCl buffer, pH 7.4 in the presence of ThT (40 µM) or ANS (50 µM). Fluorescence of ThT (Ex 440 nm, Em 485 nm), and ANS (Ex 355 nm, Em 480 nm) was measured every 10 min. Data from triplicates were averaged and fitted with a Boltzmann sigmoidal curve. (E) Kinetic parameters extracted from to a sigmoidal Boltzmann model.

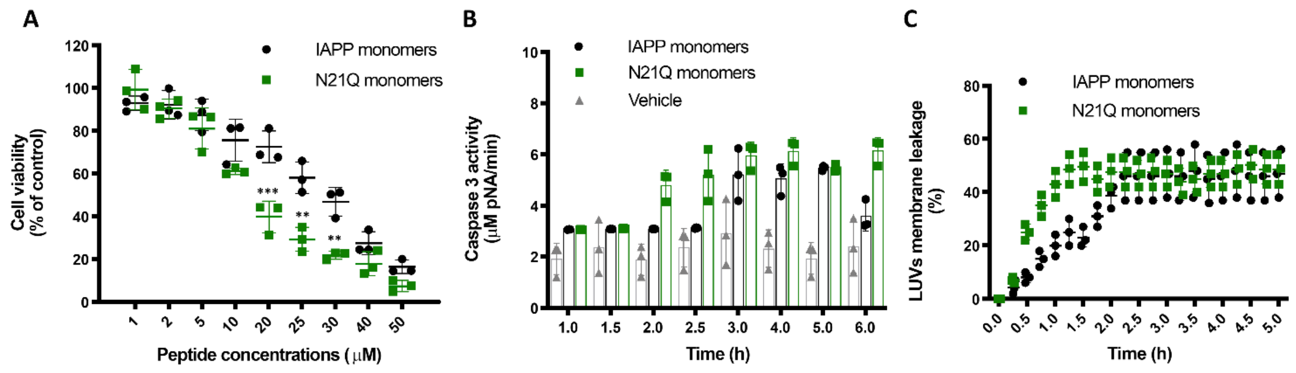

Supplementary Fig. 11 Cytotoxicity and lipid membrane perturbation induced by monomers. (A) Cytotoxicity of IAPP and N21Q monomers evaluated by measuring metabolic activity and compared to that of cells treated with the vehicle. INS-1E cells were treated for 24 h with different concentrations (0-50 μM) of IAPP and N21Q monomers. Data represent mean ± S.E.M of at least four experiments performed in triplicate. (B) Caspase-3 activation upon different incubation periods (1 to 6 h) with 50 μM IAPP and N21Q monomers. (C) Membrane leakage of 500 μM DOPC:DOPG LUVs (7:3) by 50 μM monomeric peptides.

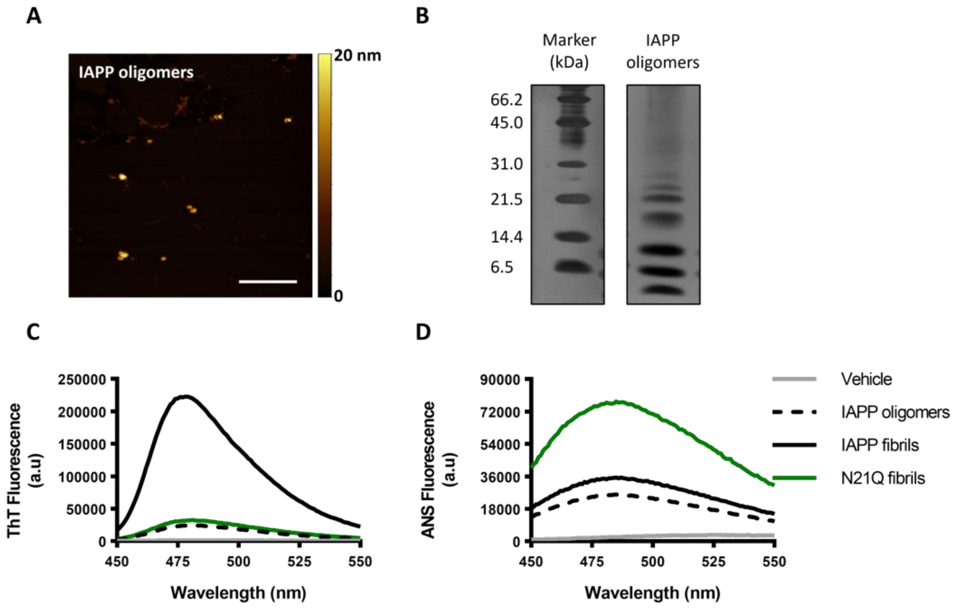

Supplementary Fig. 12 Characterization of IAPP oligomers. (A) AFM image of IAPP oligomers, scale bar: 500 nm. (B) Representative SDS-PAGE of photochemically cross-linked (PICUP) prefibrillar species. (C) ThT fluorescence of IAPP oligomers compared to IAPP and N21Q fibrils. The emission spectra of ThT (40 µM) was recorded from 450 nm to 550 nm with an excitation set at 440 nm. (D) Surface hydrophobicity of IAPP oligomers compared to IAPP and N21Q fibrils measured by ANS fluorescence. The emission spectra of ANS (100 µM) was recorded from 385 nm to 585 nm with an excitation set at 355 nm. (A-D) Oligomer mixtures were prepared from freshly dissolved monomerized peptides and incubated for 15 min at 150 µM in 20 mM Tris-HCl buffer, pH 7.4, under quiescent conditions. Assemblies were diluted to 50 µM immediately before analysis.

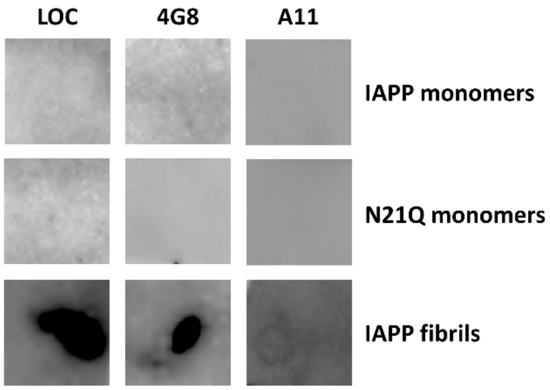

Supplementary Fig. 13 Dot-blot analysis of IAPP and N21Q monomers. Freshly dissolved monomerized peptides (20 mM Tris-HCl buffer, pH 7.4) were immediately applied to a nitrocellulose membrane. Membranes were incubated with the primary antibodies (A11, LOC, 4G8) for 2h before incubation with HRP-conjugated secondary antibody.

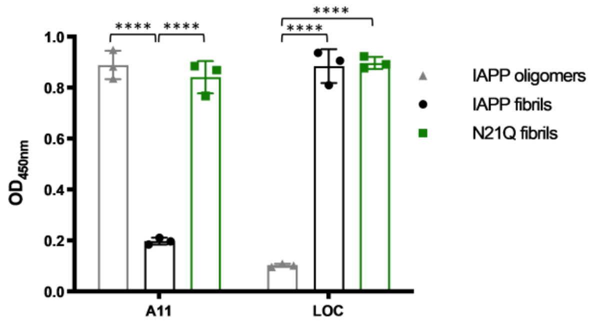

Supplementary Fig. 14 ELISA analysis of IAPP and N21Q proteospecies. Assemblies were obtained from freshly dissolved monomerized peptides incubated under quiescent conditions for 15 min (oligomers) and 48 h (fibrils) at a concentration of 150 µM in 20 mM Tris-HCl buffer, pH 7.4. High-binding ELISA plates were coated with 1.5 µM of peptide assemblies (oligomers or fibrils) overnight at 4°C. Wells were washed three times and blocked with PBS 0.05% tween-20. Coated plates were treated with conformational antibodies (1:1000; A11: anti-oligomers or LOC: anti-amyloid) for 3h at room temperature. Plates were washed and incubated with peroxidase-conjugated goat anti-rabbit IgG (1:10 000). Peroxidase signal was detected using 3,3’-5,5’-tetramethyl benzedine (TMB) and by measuring absorbance at 450 nm. Data represent mean ± S.E.M.

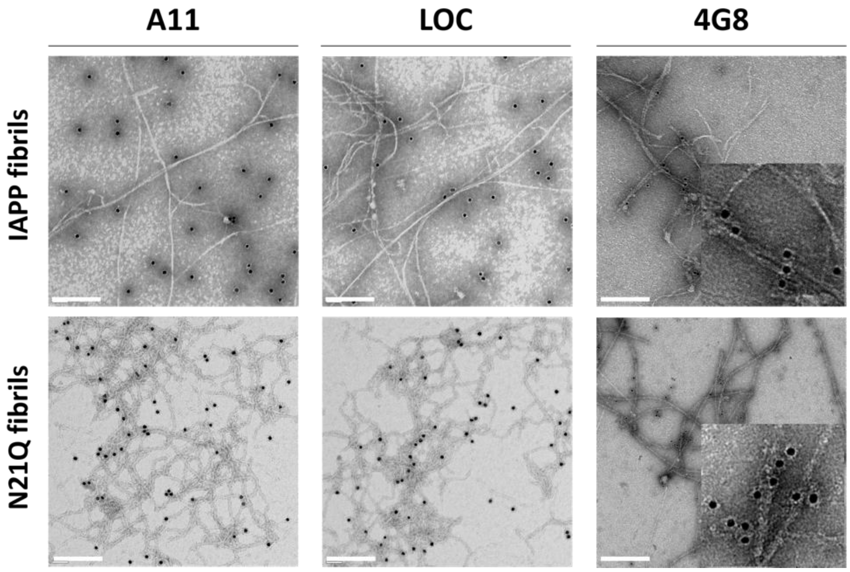

Supplementary Fig. 15 TEM-immunogold labelling of IAPP and N21Q fibrils. Immunogold electron microscopy images of IAPP and N21Q fibrils, scale bar: 500 nm. Assemblies were incubated with anti-amyloid 4G8, anti-amyloid LOC or the anti-oligomer A11. Fibrils were assembled from freshly dissolved monomerized peptides incubated under quiescent conditions for 48h at a concentration of 150 µM in 20 mM Tris-HCl buffer, pH 7.4.

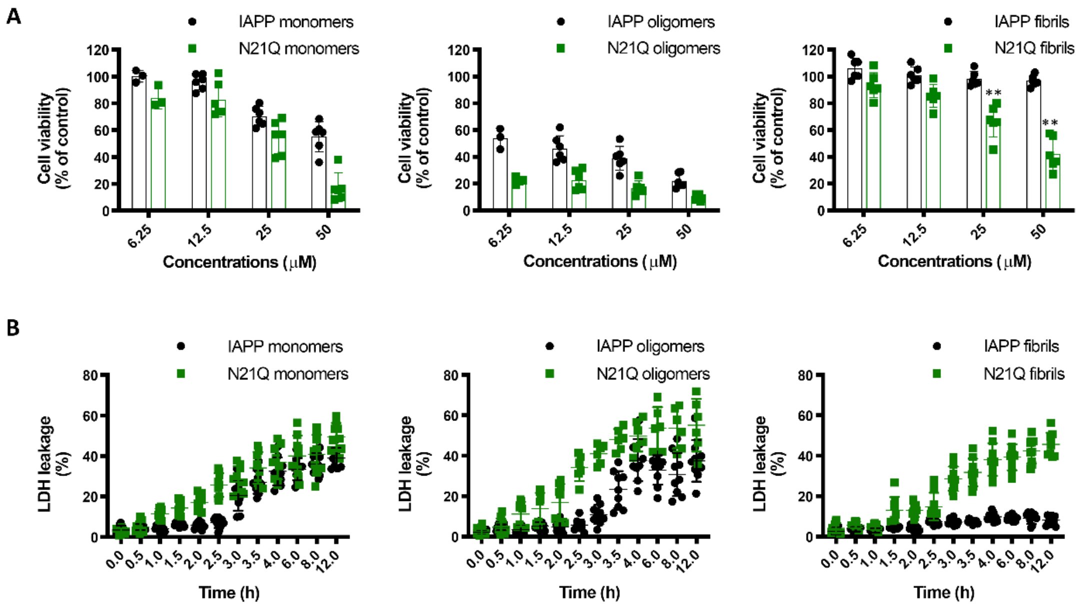

Supplementary Fig. 16 Toxicity of IAPP and N21Q assemblies on CHO K1 cells. (A) Cell viability of CHO K1 cells upon treatment with increasing peptide concentrations (6.25 to 50 µM) of WT and N21Q monomers, oligomers and fibrils. Data represent mean ± S.D. of at least four individual experiments performed in triplicate. (B) LDH release from CHO K1 cells treated with 50 µM IAPP and N21Q monomers, oligomers or fibrils for different times (0 h -12 h). (A, B) Peptide monomers, oligomers and fibrils were prepared from freshly dissolved monomerized peptides and incubated for 0 h (monomers), 15 min (oligomers) and 48 h (fibrils) at 150 µM in 20 mM Tris-HCl buffer, pH 7.4, under quiescent conditions.

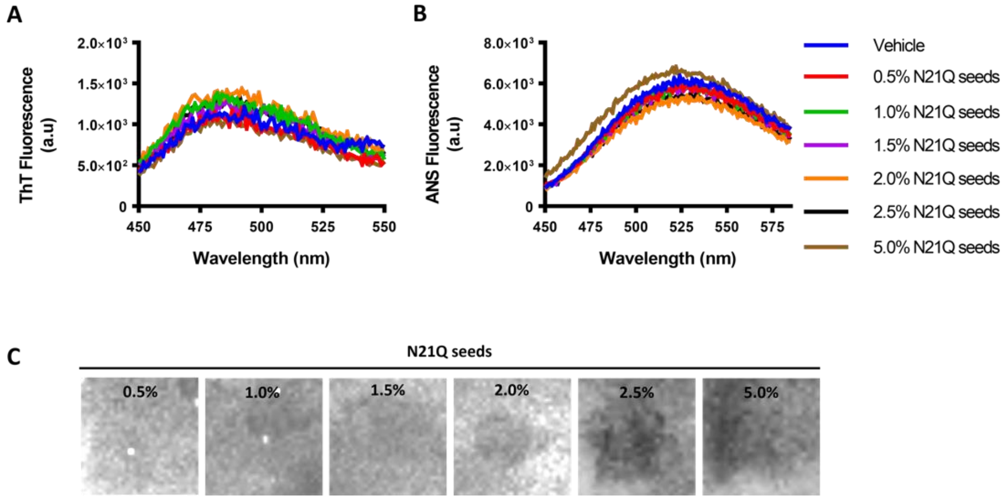

Supplementary Fig. 17 Characterization of N21Q seeds. N21Q seeds (0.25 - 2.5 µM; *i.e.* equivalent to 0.5 - 5.0 %) were characterized by (A) ThT fluorescence (Ex: 440 nm, Em: 450-550 nm), (B) ANS fluorescence (Ex: 355 nm, Em: 385-585 nm), (C) dot-blot with the anti-oligomer A11 antibody. (A-C) N21Q seeds were assembled from freshly dissolved monomerized peptide incubated under quiescent conditions for 48 h at 150 µM in 20 mM Tris-HCl buffer, pH 7.4. Seeds were diluted to reach concentrations ranging between 0.25 to 2.5 µM (0.5% to 5.0% in the seeding experiment) before analysis.

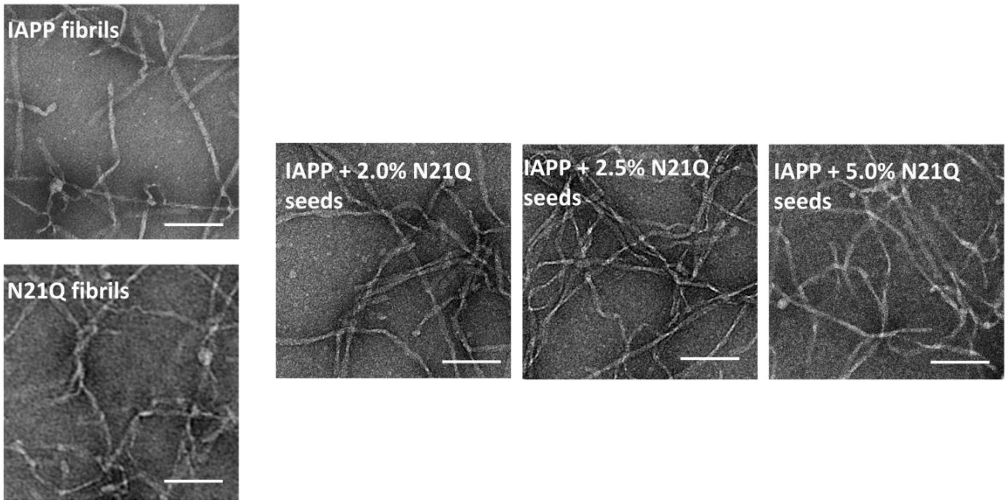

Supplementary Fig. 18 Electron microscopy images of IAPP assemblies seeded with N21Q fibrils. TEM images of WT IAPP seeded with N21Q seeds (2% - 5 mol%), scale bar: 100 nm. N21Q seeds were grown for 48 h (quiescent conditions, 150 µM in 20 mM Tris-HCl buffer, pH 7.4) before being isolated and used to seed amyloid formation of IAPP under quiescent conditions for 48 h at 150 µM in 20 mM Tris-HCl buffer, pH 7.4. Assemblies were diluted to 50 µM immediately before TEM analysis.

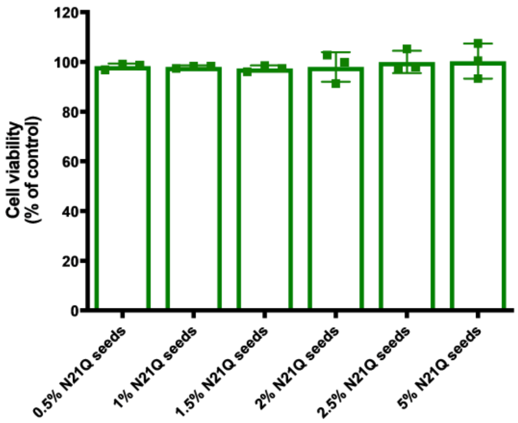

Supplementary Fig. 19 Cytotoxicity of N21Q fibrillar seeds at low concentrations. Toxicity of N21Q seeds alone (0.5% - 5.0%; 0.25 to 2.5 μM) was evaluated by measuring the metabolic activity of INS-1E cells. Data represent mean ± S.E.M of at least four experiments performed in triplicate. Fibrils were assembled from freshly dissolved monomerized N21Q peptide incubated under quiescent conditions for 48 h at a concentration of 150 μM in 20 mM Tris-HCl buffer, pH 7.4. Seeds were diluted to 0.25 to 2.5 μM (0.5% to 5.0 mol%) before evaluating cell viability.

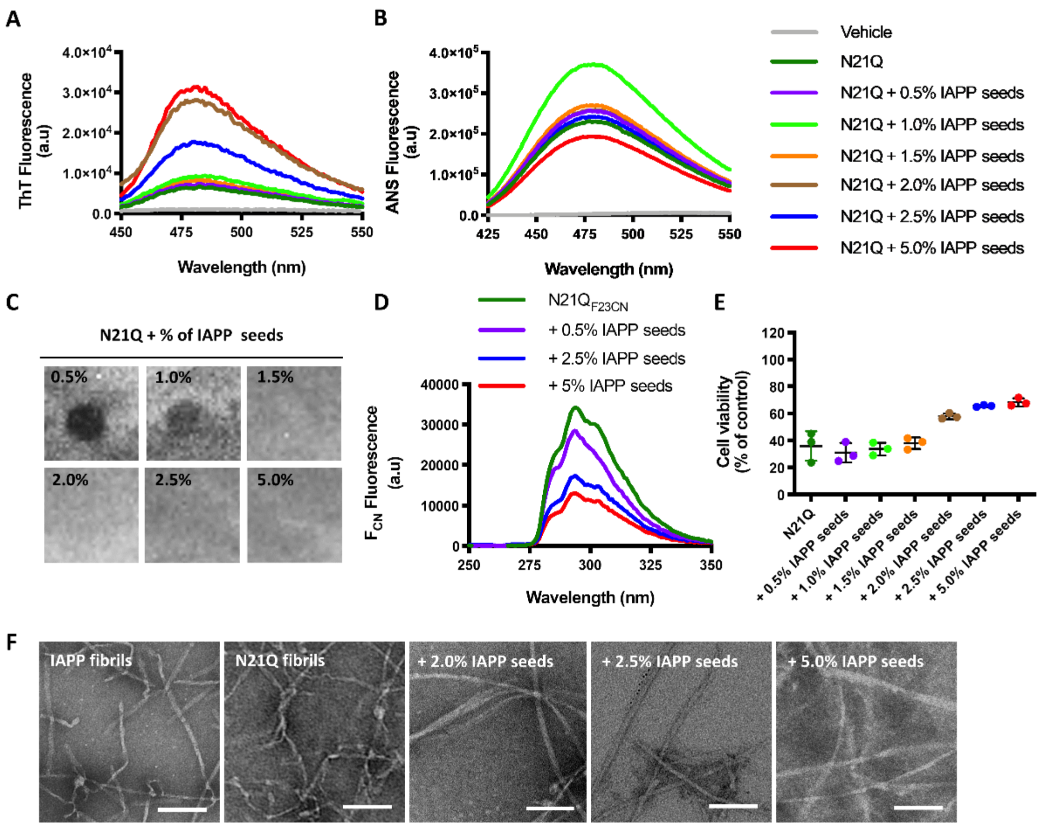

Supplementary Fig.20 Supplementing N21Q self-assembly with IAPP amyloid fibrils. Self-assembly of N21Q seeded with WT IAPP fibrils (0.5-5.0 mol%) was characterized by (A) ThT fluorescence, (B) ANS fluorescence, (C) dot-blot analysis with the conformational anti-oligomer A11 antibody and (D) F_{CN} fluorescence of the N21Q F23F_{CN} derivative. (E) Cell viability of INS-1E treated with N21Q fibrils seeded with WT IAPP pre-assembled fibrils. (F) TEM images of N21Q fibrils seeded with IAPP seeds (2 – 5 mol%), scale bar: 100 nm. (A-F) IAPP seeds were grown for 48 h (quiescent conditions, 150 µM in 20 mM Tris-HCl buffer, pH 7.4) before being isolated and used to seed the self-assembly of N21Q under quiescent conditions for 48 h at 150 µM in 20 mM Tris-HCl buffer, pH 7.4.
