# Supplementary material for: Identification of transmissible proteotoxic oligomer-like fibrils that expand conformational diversity of amyloid assemblies
Source: Commun Biol. 2021 Aug 5;4:939. doi: 10.1038/s42003-021-02466-7 (PMC8342456; doi:10.1038/s42003-021-02466-7)
Supplement: Supplementary file 3 — Description of Supplementary Files [file 42003_2021_2466_MOESM3_ESM.pdf]

Description of Additional Supplementary Files

File name: Supplementary Data 1

Description: All data underlying the graphs and charts.
