# Supplementary material for: Identification of transmissible proteotoxic oligomer-like fibrils that expand conformational diversity of amyloid assemblies
Source: Commun Biol. 2021 Aug 5;4:939. doi: 10.1038/s42003-021-02466-7 (PMC8342456; doi:10.1038/s42003-021-02466-7)
Supplement: Supplementary file 5 — Reporting summary [file 42003_2021_2466_MOESM5_ESM.pdf]

nature research

Corresponding author(s): Steve Bourgault

Last updated by author(s): Jun 26, 2021

Reporting Summary

Nature Research wishes to improve the reproducibility of the work that we publish. This form provides structure for consistency and transparency in reporting. For further information on Nature Research policies, see our Editorial Policies and the Editorial Policy Checklist.

Statistics

For all statistical analyses, confirm that the following items are present in the figure legend, table legend, main text, or Methods section.

n/a	Confirmed	
☐	☒	The exact sample size (*n*) for each experimental group/condition, given as a discrete number and unit of measurement
☐	☒	A statement on whether measurements were taken from distinct samples or whether the same sample was measured repeatedly
☐	☒	The statistical test(s) used AND whether they are one- or two-sided<br>*Only common tests should be described solely by name; describe more complex techniques in the Methods section.*
☒	☐	A description of all covariates tested
☒	☐	A description of any assumptions or corrections, such as tests of normality and adjustment for multiple comparisons
☒	☐	A full description of the statistical parameters including central tendency (e.g. means) or other basic estimates (e.g. regression coefficient) AND variation (e.g. standard deviation) or associated estimates of uncertainty (e.g. confidence intervals)
☒	☐	For null hypothesis testing, the test statistic (e.g. *F*, *t*, *r*) with confidence intervals, effect sizes, degrees of freedom and *P* value noted<br>*Give P values as exact values whenever suitable.*
☒	☐	For Bayesian analysis, information on the choice of priors and Markov chain Monte Carlo settings
☒	☐	For hierarchical and complex designs, identification of the appropriate level for tests and full reporting of outcomes
☒	☐	Estimates of effect sizes (e.g. Cohen's *d*, Pearson's *r*), indicating how they were calculated

Our web collection on statistics for biologists contains articles on many of the points above.

Software and code

Policy information about availability of computer code

Data collection	No software was used
Data analysis	ImageJ software (version 1.52)<br>Grams/AI 8.0 software<br>Prism 8 software

For manuscripts utilizing custom algorithms or software that are central to the research but not yet described in published literature, software must be made available to editors and reviewers. We strongly encourage code deposition in a community repository (e.g. GitHub). See the Nature Research guidelines for submitting code & software for further information.

Data

Policy information about availability of data

All manuscripts must include a data availability statement. This statement should provide the following information, where applicable:

- Accession codes, unique identifiers, or web links for publicly available datasets
- A list of figures that have associated raw data
- A description of any restrictions on data availability

The data supporting the findings of this manuscript are available from the corresponding authors upon reasonable request.

Field-specific reporting

Please select the one below that is the best fit for your research. If you are not sure, read the appropriate sections before making your selection.

☒ Life sciences ☐ Behavioural & social sciences ☐ Ecological, evolutionary & environmental sciences

For a reference copy of the document with all sections, see nature.com/documents/nr-reporting-summary-flat.pdf

Life sciences study design

All studies must disclose on these points even when the disclosure is negative.

Sample size	No sample-size calculation was performed. The sample-sizes were equal or larger than 3 in all cases. No method was used to determine the sample size. The sample size was determined for each experiment based on previous experiments available in the literature with the same protocol/setup and from previous studies of our group. All data referred as significant has $p < 0.05$, assuming a normal distribution.
Data exclusions	No data were excluded from the analysis.
Replication	All attempts at replication were successful.
Randomization	This is not relevant to our study.
Blinding	Blinding is not relevant to our study.

Reporting for specific materials, systems and methods

We require information from authors about some types of materials, experimental systems and methods used in many studies. Here, indicate whether each material, system or method listed is relevant to your study. If you are not sure if a list item applies to your research, read the appropriate section before selecting a response.

Materials & experimental systems

n/a	Involved in the study	
☐	☒	Antibodies
☐	☒	Eukaryotic cell lines
☒	☐	Palaeontology and archaeology
☒	☐	Animals and other organisms
☒	☐	Human research participants
☒	☐	Clinical data
☒	☐	Dual use research of concern

Methods

n/a	Involved in the study	
☒	☐	ChIP-seq
☒	☐	Flow cytometry
☒	☐	MRI-based neuroimaging

Antibodies

Antibodies used	1. Anti-amyloid fibrils LOC (rabbit polyclonal antibody, Sigma Aldrich, cat. #: AB2287, lot #: 3082480)<br>2. Anti-amyloid fibrils 4G8 (mouse polyclonal antibody, BioLegend, cat. #: 800701, lot #: B249634)<br>3. Anti-oligomer A11 (rabbit polyclonal antibody, Fisher Scientific, cat. #: AHB0052, lot #: TL276118)
Validation	All validation statements taken form suplliers website:<br>1. The anti-amyloid fibrils LOC antibody is validated for use in IP, IC, IH, ELISA, WB, DB for the detection of Amyloid Fibrils LOC.<br>2. The anti-amyloid fibrils 4G8 antibody is verified by ELISA and passed the quality test by IHC-P.<br>3. The A11 antibody recognizes amino acid sequence-independent oligomers of proteins or peptides. A11 does not recognize monomers or mature fibers of proteins or peptides. A11 recognizes oligomeric species of several other amyloidogenic polypeptides including AB42, human insulin, prion, polyglutamine, lysozyme, alpha-synuclein, IAPP and yeast prion Sup35.

Eukaryotic cell lines

Policy information about cell lines

Cell line source(s)	- INS-1 rat insulinoma cell line (Sigma Aldrich, cat. #: SCC207, https://www.sigmaaldrich.com/catalog/product/mm/scc207?lang=fr&region=CA)<br>- Chinese hamster ovary cell line, CHO-K1 (ATCC® CCL61™, https://www.atcc.org/products/all/CCL-61.aspx)
Authentication	None of the cell lines were authenticated.
Mycoplasma contamination	Cell lines were not tested for mycoplasma contamination recently. Last test was performed in 2019.

Commonly misidentified lines<br>(See ICLAC register)	Not applicable.
